# Characterization of human herpesvirus 6A/B U94 as ATPase, helicase, exonuclease and DNA-binding proteins

**DOI:** 10.1093/nar/gkv503

**Published:** 2015-05-20

**Authors:** Frédéric Trempe, Annie Gravel, Isabelle Dubuc, Nina Wallaschek, Vanessa Collin, Shella Gilbert-Girard, Guillaume Morissette, Benedikt B. Kaufer, Louis Flamand

**Affiliations:** 1 Division of Infectious Disease and Immunity, CHU de Québec Research Center, Quebec city, Quebec G1V 4G2, Canada; 2 Institut für Virologie, Freie Universität Berlin, Berlin 14163, Germany; 3 Department of microbiology, infectious disease and immunology, Faculty of Medicine, Université Laval, Quebec city, Québec,G1V 0A6 Canada

## Abstract

Human herpesvirus-6A (HHV-6A) and HHV-6B integrate their genomes into the telomeres of human chromosomes, however, the mechanisms leading to integration remain unknown. HHV-6A/B encode a protein that has been proposed to be involved in integration termed U94, an ortholog of adeno-associated virus type 2 (AAV-2) Rep68 integrase. In this report, we addressed whether purified recombinant maltose-binding protein (MBP)-U94 fusion proteins of HHV-6A/B possess biological functions compatible with viral integration. We could demonstrate that MBP-U94 efficiently binds both dsDNA and ssDNA containing telomeric repeats using gel shift assay and surface plasmon resonance. MBP-U94 is also able to hydrolyze adenosine triphosphate (ATP) to ADP, providing the energy for further catalytic activities. In addition, U94 displays a 3′ to 5′ exonuclease activity on dsDNA with a preference for 3′-recessed ends. Once the DNA strand reaches 8–10 nt in length, the enzyme dissociates it from the complementary strand. Lastly, MBP-U94 compromises the integrity of a synthetic telomeric D-loop through exonuclease attack at the 3′ end of the invading strand. The preferential DNA binding of MBP-U94 to telomeric sequences, its ability to hydrolyze ATP and its exonuclease/helicase activities suggest that U94 possesses all functions required for HHV-6A/B chromosomal integration.

## INTRODUCTION

The human beta-herpesvirus subfamily is composed of four viruses: human cytomegalovirus (CMV), human herpesvirus (HHV)-6A (HHV-6A), HHV-6B and HHV-7. Based on epidemiological, biological and molecular characteristics, HHV-6A and HHV-6B were recently recognized as distinct viruses rather than viral variants (1,2). HHV-6B is the etiological agent of the sixth disease (roseola infantum) (3) and with virus reactivation being associated with various problems including encephalitis in hematopoietic stem cell transplantation, especially when using cord blood as the source of stem cells (4). The epidemiology and disease associations with HHV-6A are less clear.

Intriguingly, HHV-6A and HHV-6B have been shown to integrate their genomes into the telomeres of human chromosomes, while most herpesviruses maintain their genomes as extra chromosomal circular episomes in the nucleus of infected cells during latency. HHV-6 chromosomal integration was first discovered in the mid 1990's, when Luppi and colleagues identified the presence of integrated HHV-6 DNA into the chromosomes of freshly isolated peripheral blood mononuclear cells (PBMC) (5–7). Several independent investigators have confirmed these findings (reviewed in (8,9)). This integration can occur in both somatic and germ cells. Chromosomal integration into germ cells can result in individuals that harbor HHV-6A/B in every single cell of their body. Fifty percent of their descendants will inherit this condition, which we referred to as inherited chromosomally-integrated HHV-6 (iciHHV-6). It is estimated that between 0.5–1% of the world population are iciHHV-6+ and carry an integrated copy of the HHV-6A/B genome in every cell of their body (8–10). Both HHV-6 species can integrate, with HHV-6B integration representing two-thirds of iciHHV-6 cases reported so far (8). Recent reviews address some of the biological issues and concerns associated with iciHHV-6 (8,10–12). Work by Arbuckle *et al*. indicates that integration also occurs in cell infected *in vitro*, indicating that HHV-6A/B integration can be quite common and not only occurs in the germ line (13). The underlying mechanisms of HHV-6A/B integration remain completely unknown.

The HHV-6A/B genome consists of a single unique component (U) (∼145 kbp) flanked by identical direct repeats (DR) (∼9 kbp) (14–17) that are flanked by the cleavage and packaging signals pac1 and pac2 (18,19). Adjacent to the pac2 sequences are arrays of TTAGGG repeats that are identical to the human telomeric repeat sequence (TRS). In addition, imperfect TRS referred as het(TAACCC)_n_ are adjacent to pac1 (16,19). Intriguingly, TRS are found in several lymphotropic herpesviruses belonging to the α-herpesvirinae (20–23), β-herpesvirinae (14,16,24,25) and γ-herpesvirinae (26). While HHV-6A/B (5–7) and the oncogenic Marek's disease virus (MDV) (27,28) have been shown to integrate into host chromosomes, it remains unclear if the other TRS-containing virus including HHV-7, a close relative of HHV-6A/B, are capable of integration (29). The TRS in the MDV genome have been shown to facilitate integration into host telomeres, a process that is essential for efficient pathogenesis and tumor formation (30). It has been hypothesized that homologous recombination events between the viral TRS and host telomeres results in viral integration (8,9). All integration sites identified so far localize the HHV-6A/B genome in the telomeric/sub-telomeric regions (1q44, 9q34.3, 10q26, 11p15.5, 17p13.3, 18p11.3, 18q23, 19q13.4 and 22q13.3) (reviewed in (8)). One factor that is thought to play a role in HHV-6A/B integration is the putative integrase U94, coding for 490 amino acid protein homologous (24% identity) to the AAV Rep78/68, a non-structural protein essential for AAV integration within chromosomes 19 (31–33). U94 is unique to HHV-6A and HHV-6B with no ortholog in other human herpesviruses. The U94 protein is identical between isolates of the same species ((34) and Trempe and Flamand unpublished data) and shares a 97% amino acid identity between HHV-6A and HHV-6B species (15,17). The only other known herpesvirus having an open reading frame (ORF) (r127) homologous to AAV Rep78/68 is rat CMV (35). Rat CMV does not harbor TRS and it remains unknown if this virus integrates into host chromosomes. HHV-6B U94 mRNA and protein are expressed at very low level during infection (34) and ectopic expression of U94 inhibits HHV-6 lytic replication (36), angiogenesis and lymphomagenesis in cancer cells (37). U94 demonstrates single-stranded DNA binding properties (36,38,39) and is reported to interact with TATA-binding protein (39). However, the molecular functions of HHV-6A/B U94 remain largely unknown.

In this report, we determined the molecular functions of U94 including those of Rep78/68 prove essential for AAV parvoviral integration (40). We could demonstrate that HHV-6A/B U94 possess DNA-binding, exonuclease and helicase-ATPase activities that are compatible with integration of HHV-6 into host telomeres.

## MATERIALS AND METHODS

### Prokaryotic expression vectors

The U94 ORF was amplified by polymerase chain reaction (PCR) using HHV-6A GS and HHV-6B Z29 viral DNA as template. PCR reactions (50 μl) contained the following reagents: 1× reaction buffer, 10 ng of viral DNA, 0.4 μM of each primer, 200 μM dNTP and 2.5 units of polymerase Expand High Fidelity^PLUS^ PCR System (Roche Diagnostics, Laval, Canada). Products were amplified as follow: 94°C for 2 min, ten cycles of [30 s at 94°C, 30 s at 50°C and 2 min at 72°C], 20 cycles of [30 s at 94°C, 30 s at 50°C and 2 min + 10 s per cycle at 72°C] to finish with a 7 min at 72°C. The following primer pair was used: U94 forward 5′-CGCGGATCCTTTTCCATAATA AATCC-3′ and U94 reverse 5′-CGCAAGCTTTTATAAAATTTT(C/T)GG-3′ (Integrated DNA Technologies, Coralville, IA, USA). To facilitate cloning, restriction sites (underlined) were included at the 5′-end of each primer. PCR products were run on a 0.8% agarose gel and extracted with the QIAquick Gel extraction kit (Qiagen, Toronto, Canada). The purified PCR product was digested with BamHI (New England Biolabs Inc., Whitby, Canada) and HindIII (Roche Diagnostics) and ligated into BamHI/HindIII digested pMAL-c2 vector (NEB). Sequencing confirmed that U94 was ligated in-frame with maltose-binding protein (MBP). The pMAL-c2-Rep68 plasmid was obtained from R.A. Owens (National Institute of Diabetes and Digestive and Kidney Diseases, National Institutes of Health, Bethesda, MD, USA) (41).

### Production and purification of recombinant proteins

BL21-RIL *Escherichia coli* bacteria (Agilent Technologies Canada Inc., Mississauga, Canada) were transformed with 100 ng of pMAL-c2 or fusion construct (MBP-U94A, MBP-U94B, MBP-Rep68). Bacteria were plated on LB agar with 100 μg/ml of ampicillin and 25 μg/ml of chloramphenicol and grown at 37°C overnight. The next day, bacterial pre-cultures were used to inoculate (1:100 dilution) two 2-l flasks containing 500 ml of LB and antibiotics. Bacteria were grown at 37°C with agitation (250 rpm) for ∼3 h until the optical density at 600 nm (D.O._600 nm_) reached 0.4–0.6. Protein expression was induced by adding 0.3 mM of isopropyl β-D-1-thiogalactopyranoside (IPTG) (Fisher Scientific, Ottawa, Canada) to each flask. The cultures were switched to room temperature and incubated overnight with agitation. Bacterial cultures were centrifuged at 4000 × *g* using a Sorvall RC 3C PLUS centrifuge (rotor H6000A) for 20 min at 4°C. Bacteria were resuspended in a total of 20 ml of resuspension buffer (20 mM Tris–HCl pH 7.5, 1 mM DTT, 1 mM EDTA, 200 mM NaCl) and lysed by sonication (2.5 min at output 3.5) on ice using a Sonifier 450 (Branson, Danbury, CT, USA). Samples were centrifuged at 9000 × *g* in an Avanti J-E with JA-20 rotor (Beckman Coulter, Mississauga, Canada) for 30 min at 4°C and supernatants were diluted 1:10 with resuspension buffer. MBP recombinant proteins were purified using MBPTrap™ HP 1 ml column (GE Healthcare, Uppsala, Sweden) and ÄKTAprime plus purification system (GE Healthcare). The purified proteins were dialyzed two times against 1 l of phosphate buffered saline (PBS) using Slide-A-Lyzer Dialysis cassettes with 10K molecular weight cut off (Thermo Scientific, Rockford, IL, USA). Protein concentrations were determined using the bicinchoninic acid assay (BCA) (Thermo Scientific). All proteins were aliquoted and stored at −80°C.

### Western blot and coomassie blue staining

Purified recombinant proteins were analyzed by western blot and coomassie blue staining. MBP-U94A, MBP-U94B, MBP-Rep68 and MBP proteins were loaded and electrophoresed through 7.5% polyacrylamide-sodium dodecyl sulphate (SDS) gels. Gels were stained using coomassie blue solution (0.025% Coomassie Brillant Blue R-250, 40% methyl alcohol, 10% glacial acetic acid and 50% water), destained and dried. For western blots, migrated proteins were transferred on PVDF membranes (Immobilon^®^ Millipore, Etobicoke, Canada). Membranes were blocked with a solution of 5% non-fat milk in TBS containing 0.01% Tween 20 (TBST). Membranes were probed with a polyclonal rabbit anti-MBP diluted 1:1000 (Applied Biological Materials Inc., Richmond, Canada) for one hour at room temperature. After three 5-min washes with TBST, the secondary antibody, a peroxidase-labeled goat anti-rabbit IgG antibody diluted 1:10000 (Jackson Immuno Research Laboratories, Inc., West grove, AR, USA) was added for 1 h at room temperature. After three 5-min washes with TBST, labeled proteins were visualized by enhanced chemiluminescence (Western Lightning^®^ Plus-ECL, PerkinElmer, Woodbridge, Canada).

### Surface plasmon resonance assay (SPR)

All surface plasmon resonance assay (SPR) experiments were conducted using the ProteOn XPR36 apparatus (Bio-Rad, Mississauga, Canada). Biotin-labeled DNA oligonucleotides were attached to the surface of a neutravidin-coated NLC chip (Bio-Rad). The neutravidin-coated NLC chip (Bio-Rad) was preconditioned by injecting a 1 M NaCl and 50 mM NaOH solution in the two directions (horizontally and vertically). Biotinylated-oligonucleotides (Human TRS G-rich, human TRS C-rich, HHV-6 TRS C-rich, random sequence, HHV-6 TRS and ΔITR AAVS1 Rep binding site (AAV-2)) were diluted to 5 × 10^−5^ μg/μl in PBS containing 0.05% Tween-20 (PBST). The equivalent of 60 Response Unit (RU) of biotinylated-oligonucleotides was attached to the NLC chip. We next blocked free neutravidin groups by injecting a PBST solution containing 1 nM biotin. The chip was then ready for protein binding analyses. Between each of these injections, a three-step regeneration (2 M NaCl, 5 mM NaOH + 0,5 M NaCl, 0,1% SDS) program was performed to remove residual binding. Binding buffer used to dilute injected proteins consisted of 20 mM NaHEPES pH 7.4, 0.1 M NaCl, 10 mM MgCl_2_, 0,1% Tween-20, 1 mg/ml BSA and 0.83 mM of adenosine triphosphate (ATP). Proteins were injected at 50 μl/min over 180 s followed by a dissociation time of 600 s. For most protein, six different concentrations were used: 800, 400, 200, 100, 50 and 0 nM.

Similar studies were also conducted using biotinylated DNA oligonucleotides containing a single CCCTAA motif. Oligonucleotides were injected at the same concentration as described above, except that various proteins concentrations were used (1, 0.5, 0.25, 0.125 or 0.0625 μM).

### Electrophoretic mobility shift assay

Ten pmoles of oligonucleotides were 5′-end-labeled using 15 μCi ATP-[γ^32^P] (Perkin Elmer) and 20 units of T4 PNK (USB Optikinase™, Affymetrix, Santa Clara, CA, USA) in a 25 μl reaction volume. After 30 min at 37°C, the reaction was stopped by incubation at 65°C for 20 min. The reaction volume was adjusted to 50 μl with sodium chloride-Tris-EDTA (STE) buffer and labeled oligonucleotides isolated using a ProbeQuant™ G-50 column (GE Healthcare). Oligonucleotides were annealed by mixing with equimolar of complementary oligonucleotides in 1× SSC buffer and heated at 100°C for 5 min, followed by gradual cooling to room temperature. In all electrophoretic mobility shift assay assays, we used 1 μg of MBP-Rep68, MBP-U94A or MBP-U94B (all approximately 95 kDa) and 0.5 μg for MBP (50 kDa) to have an equal amount of molecules per reaction. For each reaction, 20 000 cpm of labeled probe were added to proteins in a final volume of 20 μl of 1× binding buffer [30 mM HEPES-KOH pH 7.5, 7 mM MgCl_2_, 4 mM ATP, 1 mM DTT, 0.1 mg/ml of BSA and 5% glycerol] and the reaction was incubated at 37°C for 1 h. Ten microliters of 2× migration buffer [TAE 1×, 0.025% bromophenol blue, 0.025% xylene cyanol and 6% glycerol] were added to each tube and samples were loaded and electrophoresed through a non-denaturing 6% acrylamide:bis (29:1) gel. The gels were dried and exposed to X-ray films.

### ATPase assays *in vitro*

Adenosine triphosphatase (ATPase) activity was monitored by an *in vitro* assay. Reactions were carried out in a final volume of 10 μl where 0.5 μg of purified proteins were included in reaction buffer [50 mM Tris–HCl pH 8, 20 mM NaCl and 2.5 mM MgCl_2_] with or without 0.2 μg of DNA (single or double stranded) and 1.25 × 10^−10^ moles of ATP. Reactions were incubated at room temperature for 0 or 60 min and stopped by transferring tubes on ice. Samples were first treated with 20 μl of 8 M HClO_4_ for 3 min on ice. Then, the reaction was equilibrated to neutral pH by adding 60 μl of 4 M K_2_HPO_4_. Fifty ng (5 μl) of internal standard (2 Cl-AMP) were added to each tube. Finally, samples were centrifuged at 12 000 × *g* for 5 min at 4°C, the supernatants were collected and stored at −80°C until analyzed by high performance liquid chromatography (HPLC). The HPLC purification procedure represents a modified version of a previous published report (42). The column (Synergi 4 μ hydro-RP80A° 150 × 4.6 mm (Phenomenex, Terrance, CA, USA)) was refrigerated at 12°C and equilibrated in 200 mM sodium phosphate buffer pH 5.8 (solvent A). The solvent flow rate was adjusted to 1 ml/min. To determine the retention time of the various products, a standard calibrated solution containing the following products (ATP, adenosine diphosphate (ADP), adenosine monophosphate (AMP), hypoxanthine, 2 Cl-AMP) was injected before and after each experiment. The elution profiles were determined by measuring the absorbance at 260 nm. A total of 75 μl of each sample were injected into the system. Samples were eluted using (solvent B), a 0–25% methanol and 200–150 mM sodium phosphate buffer pH 5.8 linear gradient over a 7 min time lapse. After a second 7-min incubation in buffer B, the column was re-equilibrate in solvent A for 12 min.

### Assembly of synthetic telomeric D-loop

A synthetic telomeric D-loop was generated by the successive assembly of three oligonucleotides (BB, BT and INV) (Supplementary Table 1) as described (43). BB and BT contain three phosphorotioate bonds (PS) at the 3′ extremities to prevent exonuclease attack from these sites. The integrity of the structure was analyzed by gel electrophoresis and by digestion with FokI and HpaI restriction endonucleases.

### Helicase assay

Ten pmoles of probes were 5′-end labeled using 15 μCi ATP-[γ^32^P] (Perkin Elmer) and 20 units of T4 PNK in a 25 μl reaction volume. After 30 min at 37°C, the reaction was stopped by incubation at 65°C for 20 min. The reaction volume was adjusted to 50 μl with STE buffer and labeled probes isolated using a ProbeQuant™ G-50 column (GE Healthcare). Probes were annealed to various unlabeled oligonucleotides in a 1:20 ratio in 1× SSC buffer by heating at 100°C for 5 min followed by gradual cooling to room temperature. The amount of radioactive probe was determined using a scintillation counter (Perkin Elmer). The helicase assay was carried out in a 10 μl volume containing 20 000 cpm of labeled probe, 1–3 μg of the protein of interest (MBP, MBP-U94A, MBP-U94B), +/−10 mM ATP, 25 mM Tris–HCl pH 7.4, 5 mM MgCl_2_, 5 mM DTT, and 1 μg of BSA. The helicase reaction was allowed to proceed for 1 h at 37°C and stopped by adding 10 μl of 2× loading buffer (TAE 1X, 0.025% bromophenol blue, 0.025% xylene cyanol and 6% glycerol). Samples were loaded and electrophoresed through a non-denaturing 6% acrylamide:bis (29:1) gel. The gels were dried and exposed to X-ray films.

### Exonuclease assay

Oligonucleotides were 5′-end labeled using T4 PNK and annealed as described above. In some instances, the phosphodiester bonds of the two last nucleotides were substituted with phosphorotioate bonds. The exonuclease assay was carried out in a 10 μl volume containing 20 000 cpm of labeled probe, 1–3 μg of the protein of interest (MBP, MBP-U94A, MBP-U94B), 25 mM HEPES-KOH pH 7.5, 5 mM MgCl_2_, 20 mM NaCl, 1 mM DTT and 0.1 μg of BSA. The reaction was allowed to proceed for various time (1–120 min) at 37°C and stopped by adding 10 μl of 2× loading buffer (TAE 1×, 50% formamide, 0.025% bromophenol blue, 0.025% xylene cyanol and 6% glycerol). Samples were loaded onto and electrophoresed through a 16% denaturing acrylamide:bis (29:1) gel. The gels were dried and exposed to X-ray films.

## RESULTS

### Sequence alignment of HHV-6A/B U94 and AAV-2 Rep68 proteins

The U94 proteins of HHV-6A (U94A) and HHV-6B (U94B) are among the most conserved proteins between the two viral species with 97.5% amino acid identity (15,17). HHV-6 U94 can functionally complement an AAV-2 Rep68 deletion mutant, suggesting that U94 possesses some of the biological activities of Rep78/68 (40). The Rep68 domains essential for the various enzymatic activities were previously characterized (44–46). To determine if these domains are conserved in the U94 protein of HHV-6A/B, we performed sequences alignments with Rep68 (Figure S1). The overall amino acid identity between Rep68 and U94A and U94B is 24%. Several of the amino acids required for the enzymatic activities of Rep68 (red boxes) are conserved within U94A and U94B.

### DNA-binding properties of HHV-6A and HHV-6B U94 proteins

**Figure 1.**
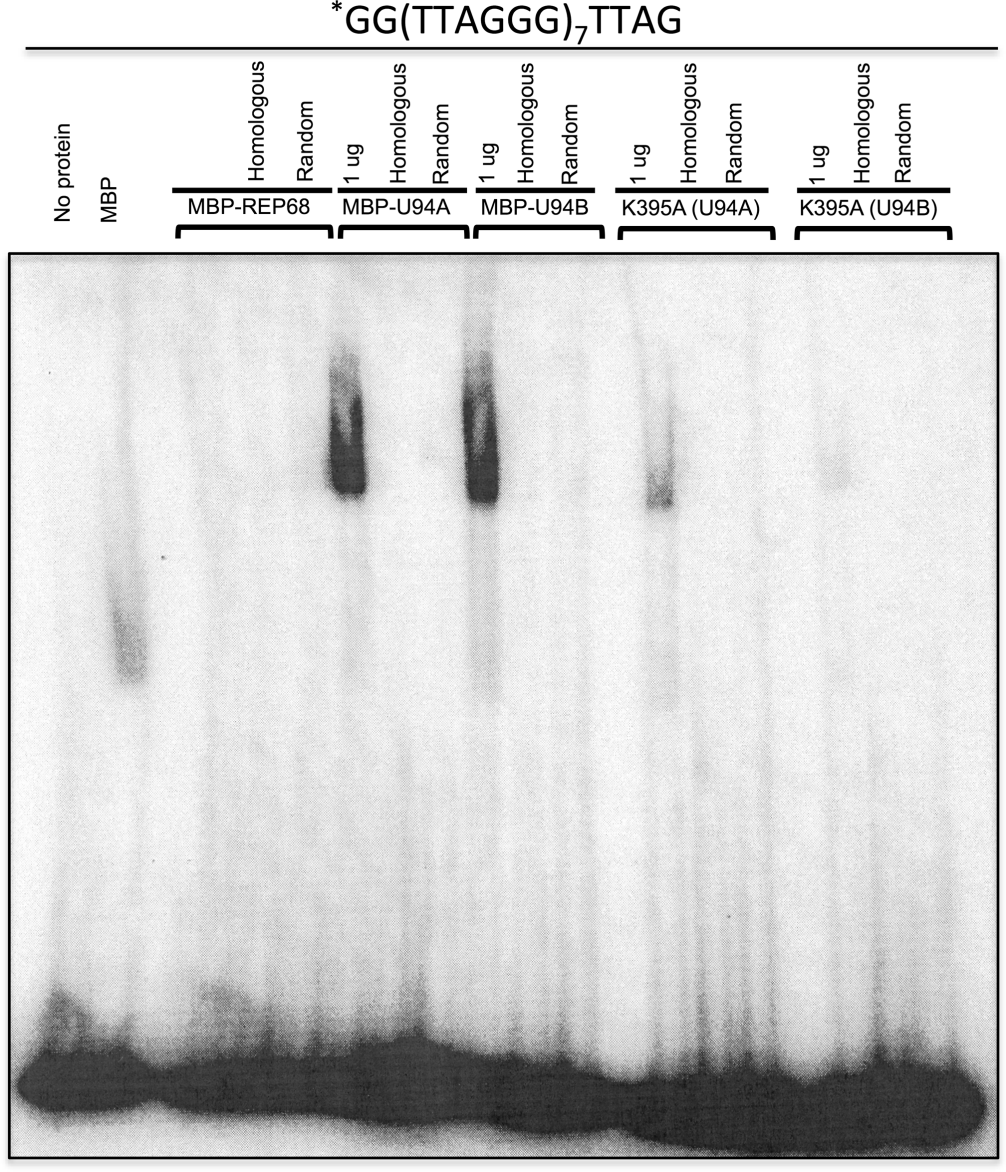
HHV-6 U94 binds single-stranded TTAGGG probes. Proteins (MBP, MBP-Rep68, MBP-U94A, MBP-U94B and MBP-U94 K395A) were incubated with ^32^P 5-end labeled (TTAGGG)_7_ probe for 30 min at 37°C in the absence or in the presence of identical (homologous) or random competitor DNA. Loading dye was added to the samples and these were electrophoresed through a non-denaturing gel. After migration the gel was dried and exposed to imaging plates. Results are representative of two independent experiments.

Previous work indicated that HHV-6 U94 binds single-stranded DNA (ssDNA) but has a low affinity for double-stranded DNA (dsDNA) (38). To determine if U94 binds ssDNA in a sequence specific manner allowing integration of HHV-6A/B into host telomeres, we determined whether U94 binds to ssDNA sequences containing the TRS motifs. First, we generated MBP-U94A and MBP-U94B expression vectors, expressed the proteins in *E. coli* and purified them by affinity chromatography (Supplementary Figure S2). Purified MBP and MBP-Rep68 were also produced and used as controls. To determine if MBP-U94 indeed binds TRS ssDNA, we performed gel shift assays using end-labeled ssDNA oligonucleotides containing the (TTAGGG)_7_ motifs (Supplementary Table 1). MBP-U94A and MBP-U94B efficiently bound to the TTAGGG motifs (Figure 1), while the MBP control protein showed minimal binding to the labeled probe. Addition of a 100-fold excess of unlabeled oligonucleotide with homologous (identical) or random sequences abrogated binding. Lysine 391 was previously shown to play an important role in the Rep68 DNA-binding activity (44). We mutated the corresponding lysine of U94 to alanine (K395A) and could demonstrate that the MBP-U94A and MBP-U94B K395A mutants were severely impaired in the binding to the (TTAGGG)_7_ ssDNA compared to MBP-U94A or MBP-U94B. Residual binding was eliminated by both homologous (identical) and random DNAs.

**Figure 2.**
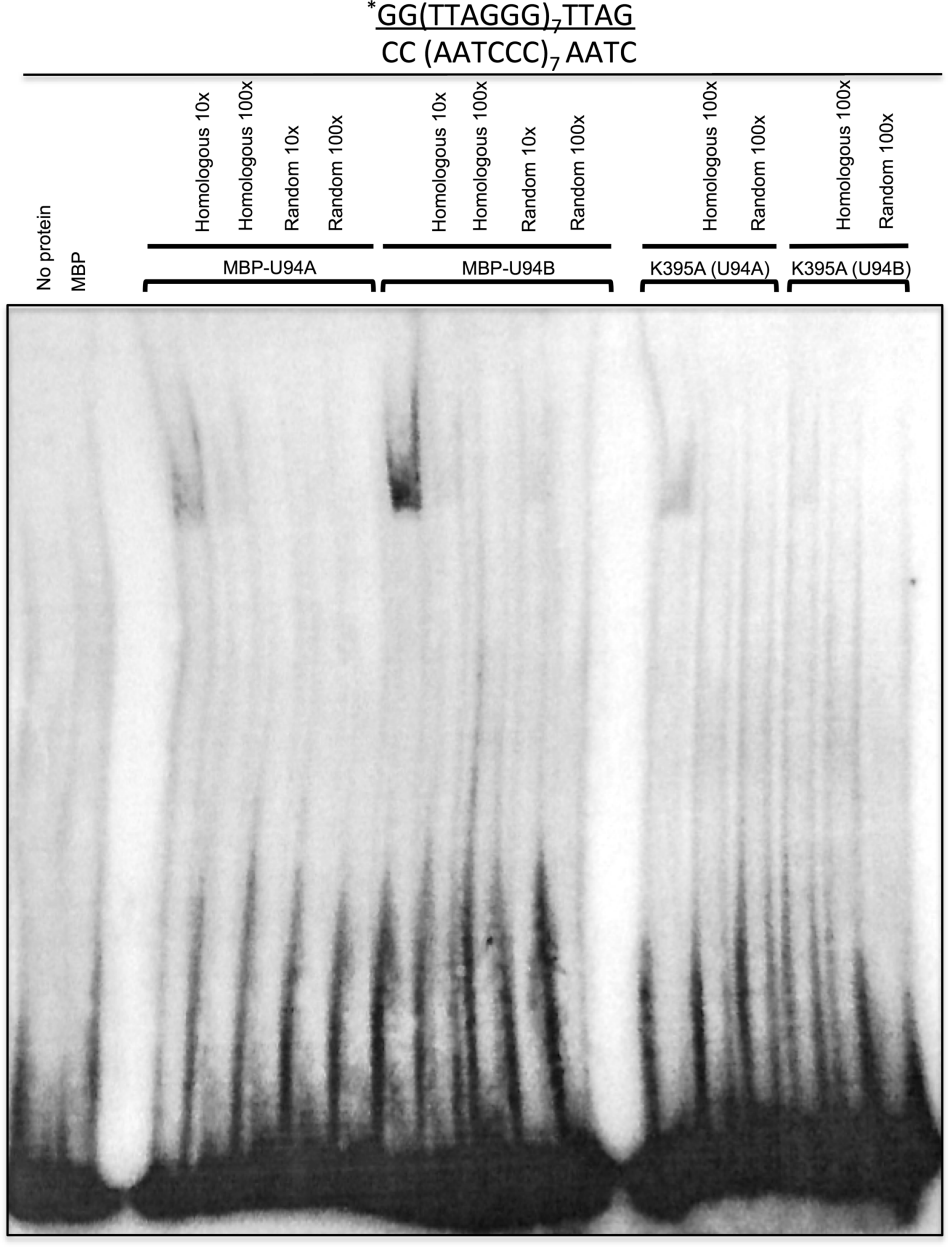
HHV-6 U94 binds double-stranded telomeric (TTAGGG/CCCTAA) probe. Proteins (MBP, MBP-Rep68, MBP-U94A, MBP-U94B and MBP-U94 K395A) were incubated with ^32^P 5-end labeled probe for 30 min at 37°C in the absence or in the presence of identical or random competitor DNA. Loading dye was added to the samples and these were separated by electrophoresis on a non-denaturing gel. After migration the gel was dried and exposed to imaging plates. Results are representative of two independent experiments.

Previous studies showed that HHV-6A/B U94 does not bind to unspecific dsDNA (36,38); however, it remained unclear if it has an affinity to dsDNA containing telomeric motifs. To address this question, we performed mobility shift assays and could demonstrate that both MBP-U94A and MBP-U94B interact with end-labeled TRS dsDNA (Figure 2), with MBP-U94B binding more efficiently than MBP-U94A. No binding was observed with the MBP control protein. As shown above, binding could be abrogated using an excess of unlabeled homologous and random dsDNAs. In addition, mutation of K395 also affected binding of MBP-U94A/B proteins, albeit this was more pronounced for MBP-U94B. Our data demonstrates that the MBP-U94 proteins from HHV-6A/B are able to bind ssDNA and dsDNA containing telomeric motifs. This binding could be eliminated by random DNAs, suggesting that MBP-U94 can also bind ssDNA and dsDNA in a sequence independent manner.

**Figure 3.**
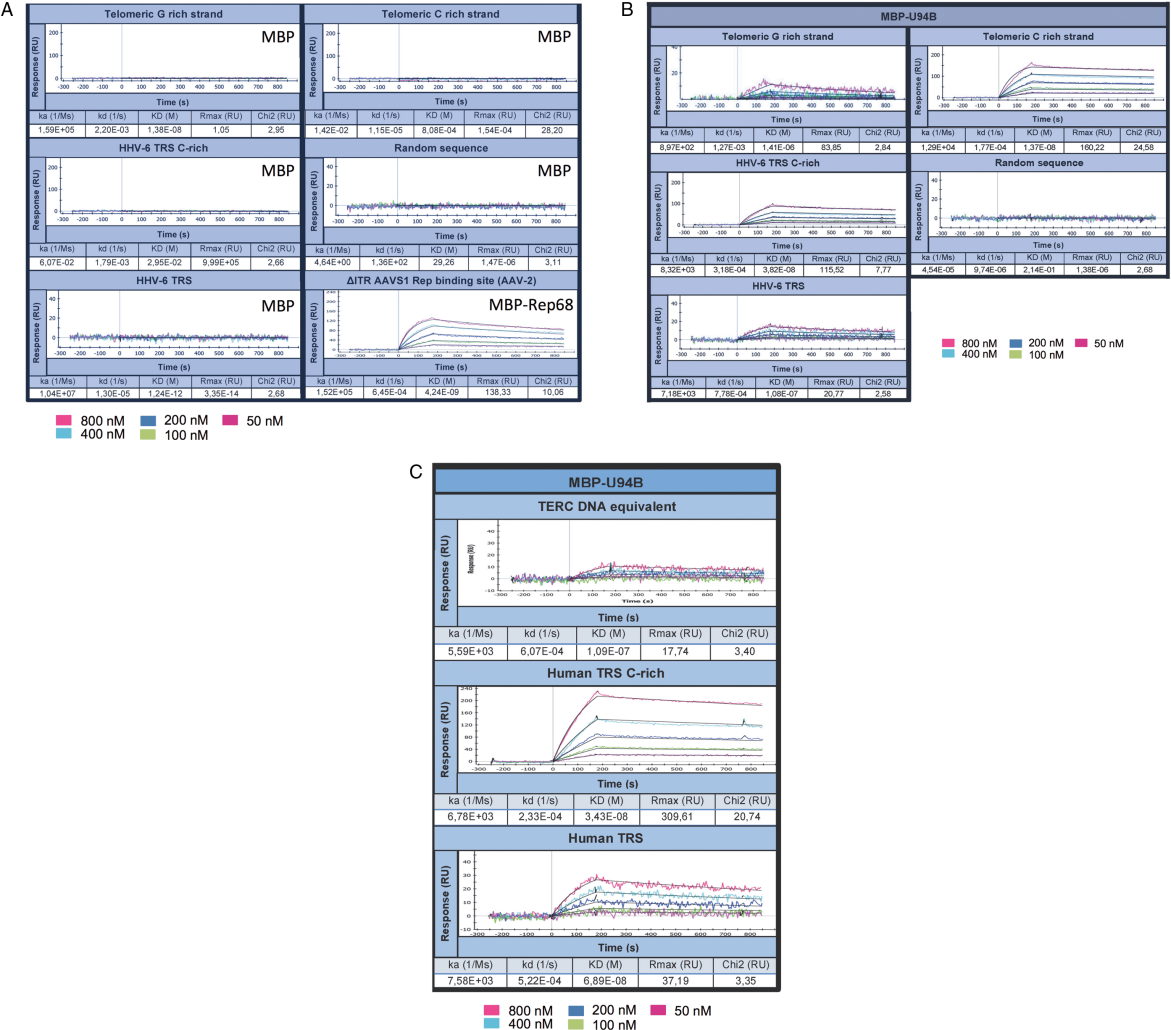
Analyses of U94 binding to DNA using SPR. Bio-sensor chips coated with various DNA oligonucleotides (Supplementary Table 1) were used to monitor the binding of MBP-U94 to the different DNAs. (**A**) Dose-response binding of MBP (negative control) and MBP-Rep68 (positive control) to various DNA substrates. (**B**) Dose-response binding of MBP-U94B to various DNA substrates. (**C**) Dose-response binding of MBP-U94B to a single CCCTAA motif (top), to 7 CCCTAA motifs (middle) and to human dsDNA telomeric motifs (bottom). Results are representative of three independent experiments.

To determine the MBP-U94 affinity toward TRS relative to unspecific DNA sequences, we performed SPR to determine the association and dissociation of MBP-U94 to the ssDNA and dsDNA in real time (oligo sequences are presented under Supplementary Table 1). MBP and MPB-Rep68 were used as negative and positive controls, respectively (Figure 3A). No binding was observed for the MBP protein, while MBP-Rep68 showed a strong interaction with its natural ligand AAV S1 dsDNA as described previously (47). We could demonstrate that MBP-U94B efficiently binds (TTAGGG)_7_ and (CCCTAA)_7_ ssDNA (Figure 3B) with a 100× greater affinity for the CCCTAA motif with a KD of 13.7 nM. To confirm that MBP-U94 also binds to the TRS sequences in the HHV-6 genome, we performed SPR using HHV-6 derived CCCTAA motifs (HHV6A/B C-rich TRS) and dsDNA (Figure 3B). We could demonstrate that MBP-U94B efficiently binds HHV-6A/B dsDNA carrying telomeric repeats. DNA binding was sequence specific as MBP-U94B did not bind a scrambled ssDNA sequence of similar length (Figure 3B). The MBP-U94B K395A mutant displayed a much lower binding affinity to ssDNA with the CCCTAA motif compared to MBP-U94B and lost all binding to dsDNA (data not shown). Similar results were obtained with MBP-U94A (data not shown). MBP-U94B binding to ssDNA was highly impaired when the number of CCCTAA repeats was reduced from 7 (Figure 3C middle panel) to 1 (Figure 3C top panel). Subsequent experiments indicated that a minimum of three CCCTAA motifs was needed to obtain the maximal MBP-U94B binding (data not shown). Finally, we could confirm that MBP-U94B also binds human dsDNA telomeric sequences (Figure 3C bottom panel).

Our results demonstrate that HHV-6A and HHV-6B U94 proteins efficiently bind ssDNA molecules with a greater affinity for the CCCTAA ssDNA strand of the telomeric repeats. Lastly, our data show for the first time that HHV-6A/B U94 proteins are able to bind dsDNA of viral and human telomeres.

### U94 proteins possess ATPase activity

**Figure 4.**
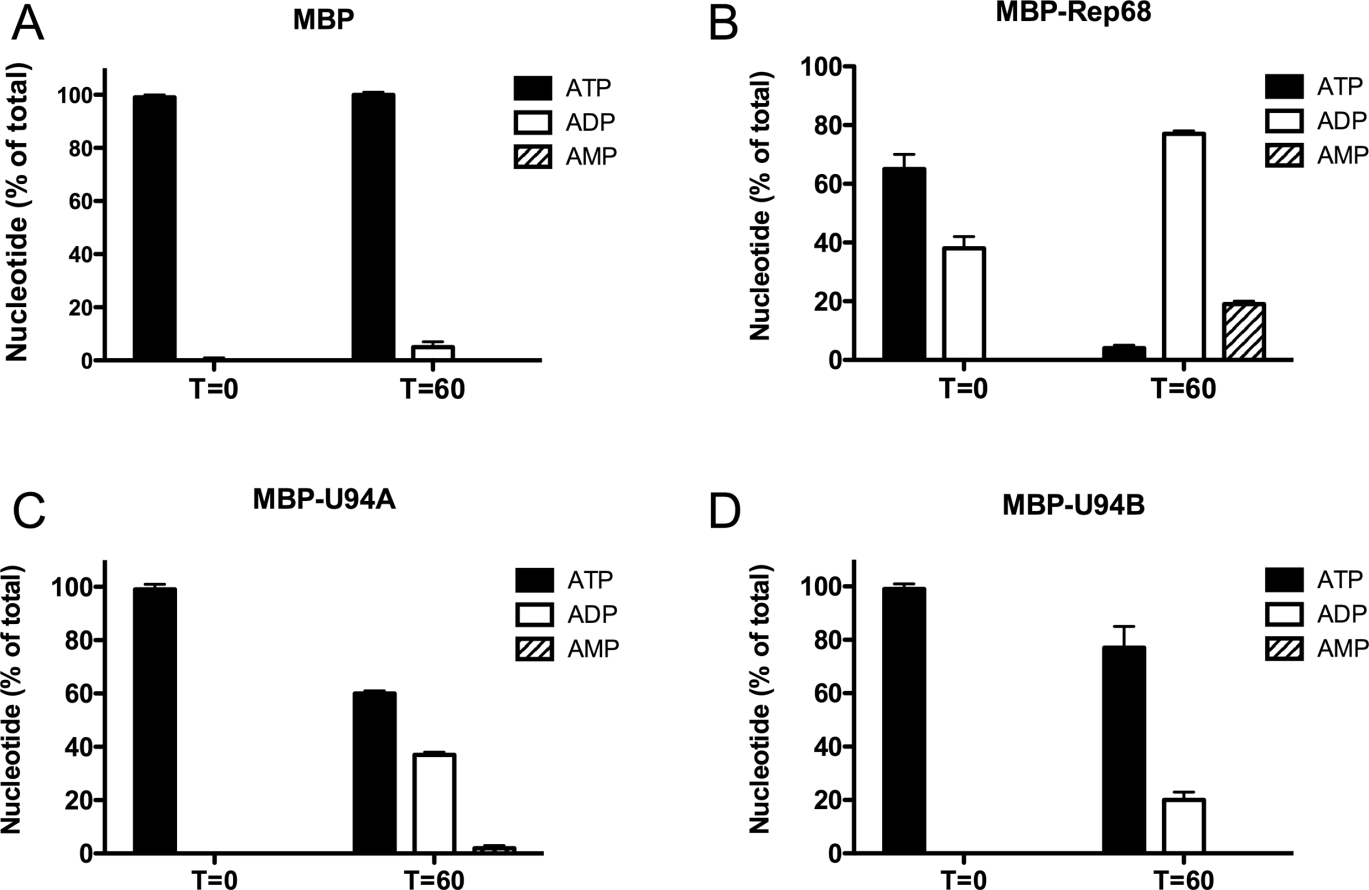
Characterization of HHV-6 U94 ATPase activity. Results of ATP, ADP and AMP hydrolysis by MBP (negative control) (**A**), MBP-Rep68 (positive control) (**B**), MBP-U94A (**C**) and MBP-U94B (**D**). Results are mean ± SD of triplicate percentages of ATP, ADP and AMP relative to the zero time point. Results are representative of three independent experiments.

We next determined the HHV-6A/B MBP-U94 ATP and ADP hydrolyzing activities by HPLC. This first series of experiments were conducted in the presence of ssDNA (TAACCC)_7_. MBP had marginal ATP hydrolysis activity (Figure 4A). As described previously, ATP and ADP hydrolysis by MBP-Rep68 was very efficient and was rapidly detected (Figure 4B). In fact, the MBP-Rep68 enzyme is very active and at *T* = 0, which reflects the interval of time between the addition of MBP-Rep68 and the time required to stop the reaction (estimated at a few seconds), partial hydrolysis of ATP into ADP is observed. MBP-U94A (Figure 4C) and MBP-U94B (Figure 4D) were also able to hydrolyze ATP. Next, we studied whether the presence of DNA was required for the ATPase activity of MBP-U94. ATP hydrolysis by MBP-Rep68, MBP-U94A and MBP-U94B occurred as efficiently in the absence of DNA (data not shown). These results indicate that the MBP-U94 proteins possess ATPase activity measured by the hydrolyzing ATP into ADP, which is independent of the presence of a DNA substrate.

### U94 proteins display helicase and exonuclease activities

**Figure 5.**
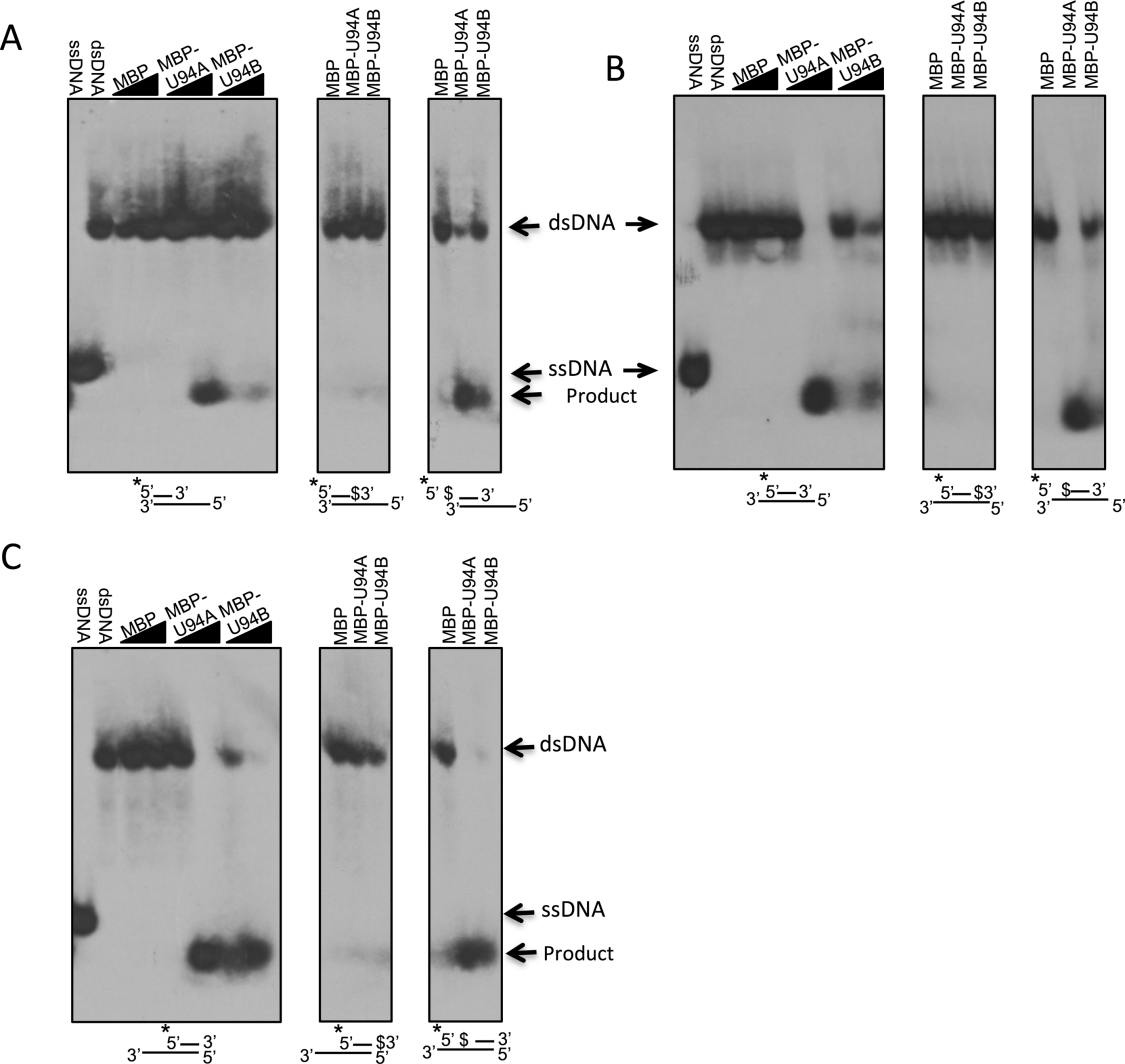
Characterization of HHV-6 U94 helicase/exonuclease activity. MBP and MPB-U94 proteins were incubated with a partial dsDNA duplexes containing either a 3′ recessed end (**A**), 5′ and 3′ recessed ends (**B**) or a 5′ recessed end (**C**). Asterisk (*) denotes the labeled strand. Proteins were also incubated with duplexes in which the last (middle panel) or first (right panel) phosphodiester bond was replaced with a phosphorotioate bond ($). After incubation, samples were analyzed by non-denaturing gel electrophoresis. Gels were dried and exposed to film. Results are representative of two independent experiments.

Unwinding of dsDNA is an energy consuming activity carried out by proteins referred to as helicases. As MBP-U94 proteins can efficiently generate energy through ATP hydrolysis, we set to determine if these proteins display helicase activity. In the first experiment, we investigated whether MBP-U94 could unwind a partial DNA duplex. A 20 nt 5′-end labeled probe was hybridized to the 3′ of a 68 nt oligonucleotide, containing eight copies of the CCCTAA telomeric motif at its 5′ end. Recombinant proteins were incubated with the partial dsDNA duplex for 1 h followed by electrophoresis on a non-denaturing gel. Both MBP-U94A and MBP-U94B were able to unwind the labeled probe in a U94 protein concentration-dependent manner, while MBP alone did not possess any helicase activity (Figure 5A). Intriguingly, the released ssDNA labeled probe migrated faster in the presence of U94, suggesting that the protein possesses an exonuclease activity. To analyze this in greater details, the last phosphodiester bond at the 3′ or 5′ end of the labeled probe was replaced with a PS, making the probe much more resistant to hydrolysis by exonucleases. The exonuclease resistant probe also protected the substrate from the helicase activity of MBP-U94 (Figure 5A-B-C, middle panel), suggesting that in the presence of a 3′ recessed end, the exonuclease activity of MBP-U94 at a blunt end is limited. When the PS bond was moved to the probe 5′ end, both MBP-U94A and MBP-U94B were able to dissociate the DNA duplex (Figure 5A–C right panel) and the labeled probe was processed by the U94 exonuclease activity resulting in a faster migration compared to the control labeled ssDNA. The observation that the 3′ phosphorothioate bond prevented strands dissociation suggests that MBP-U94 has weak helicase activity and must rely on its 3′ to 5′ exonuclease activity to reduce the probe length to ∼8–10 nt before it can be efficiently dissociated from the other strand.

We next determined the influence of probe location on MBP-U94 helicase/exonuclease activity. To do so, we generated partial DNA duplexes where the labeled probe hybridized either in the center (Figure 5B) or at the 5′ end on the 68 nt oligonucleotide (Figure 5C). Both MBP-U94A and MBP-U94B proteins were capable of separating the DNA duplex regardless of the location at which the probe was hybridized, suggesting that MBP-U94 is active against blunt and 3′ recessive ends but not against 3′ protruding ends. Every time the helicase activity was observed, the ssDNA probe migrated faster indicative of the MBP-U94 exonuclease activity. As presented above, the introduction of a PS bond at the 3′ end of the probe eliminated the helicase/exonuclease activity while the introduction of a PS bond at the 5′ end had no effect, indicating that the exonuclease activity occurs in a 3′ to 5′ direction.

**Figure 6.**
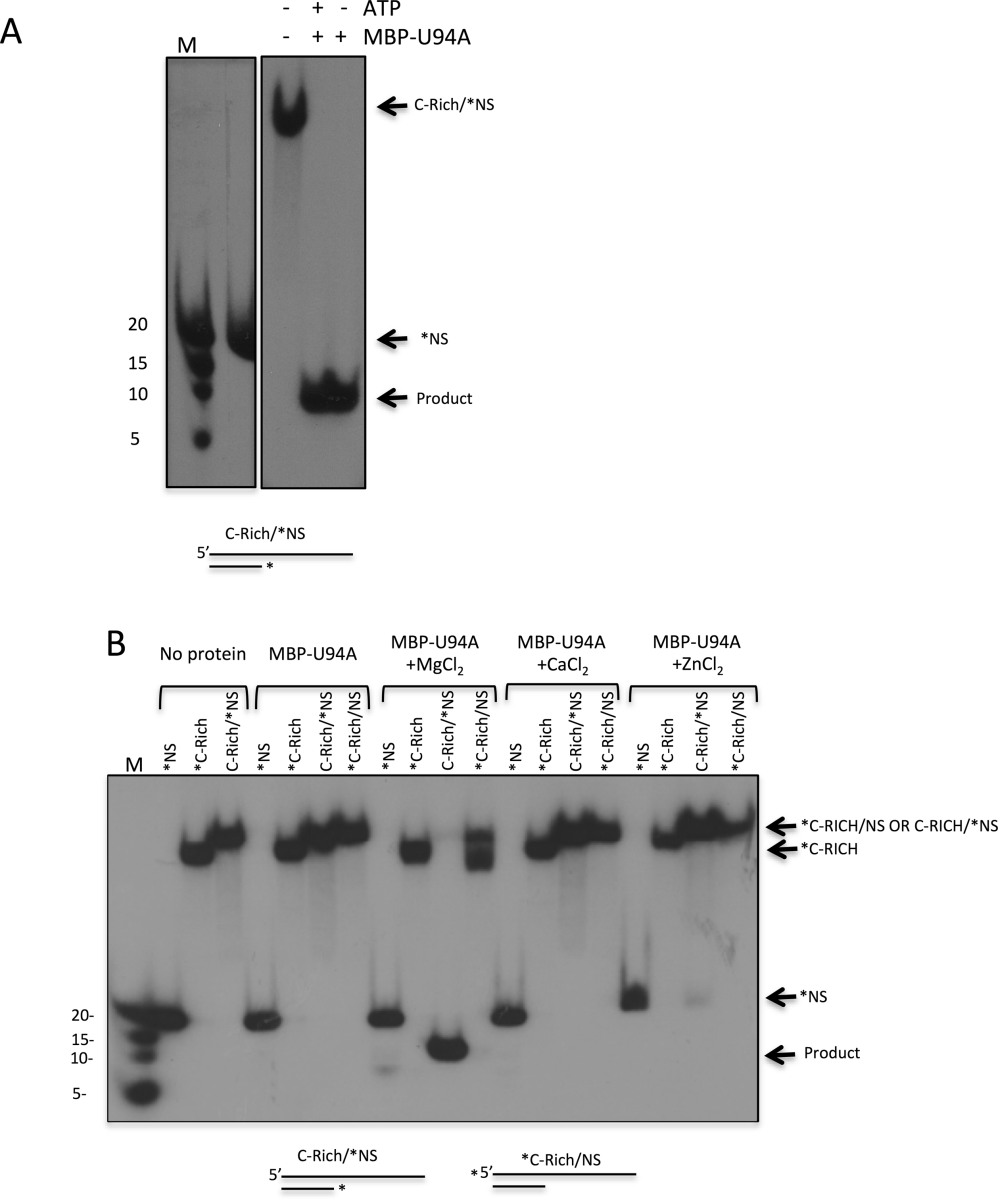
Characterization of ATP and divalent cations requirements for HHV-6 U94 helicase/exonuclease activity. (**A**) MBP-U94A was incubated with a partial dsDNA duplex (C-Rich/NS, Supplementary Table 1) in the absence or in the presence of 10 mM ATP. The asterisk (*) denotes the 5′ end-labeled strand. After incubation, samples were analyzed by non-denaturing gel electrophoresis. Gels were dried and exposed to film. (**B**) Proteins were incubated with partial dsDNA duplexes (C-Rich/NS) in the absence or in the presence of MgCl_2_, CaCl_2_ or ZnCl_2_. The asterisk denotes the 5′ end-labeled strand. After incubation, samples were analyzed by non-denaturing gel electrophoresis. Gels were dried and exposed to film. Results are representative of three independent experiments.

We next examined the ATP and divalent ion requirements for the MBP-U94 helicase activity, as helicases typically derive energy to unwind DNA duplex through ATP (or other nucleotide) hydrolysis. The DNA duplex substrate was efficiently separated by MBP-U94A in the absence or in the presence of ATP (Figure 6A), indicating that both helicase and exonuclease activities are ATP-independent. Adding apyrase, an agent that depletes ATP, to the MBP-U94 reaction had no effect on helicase and exonuclease activities, ruling out the possibility that ATP co-eluted with MBP-U94 during purification (not shown). Next, the importance of divalent cations for MBP-U94 activity (in the absence of ATP) was analyzed. In the absence of MgCl_2_, MBP-U94A has no effect on single-stranded (*NS and * C-Rich) and double-stranded probes (C-Rich/*NS and *C-Rich/NS) (Figure 6B). Addition of MgCl_2_ restored MBP-U94 activity as witnessed by the complete stripping of the NS probe from its C-Rich counterpart. Similar results were obtained when the C-Rich strand was end-labeled. When CaCl_2_ or ZnCl_2_ was used in replacement of MgCl_2_ no activity was recorded. Addition of ZnCl_2_ to MgCl_2_ also inhibited exonuclease/helicase activities of MBP-U94 (data not shown). Similar results were obtained with MBP-U94B (data not shown).

**Figure 7.**
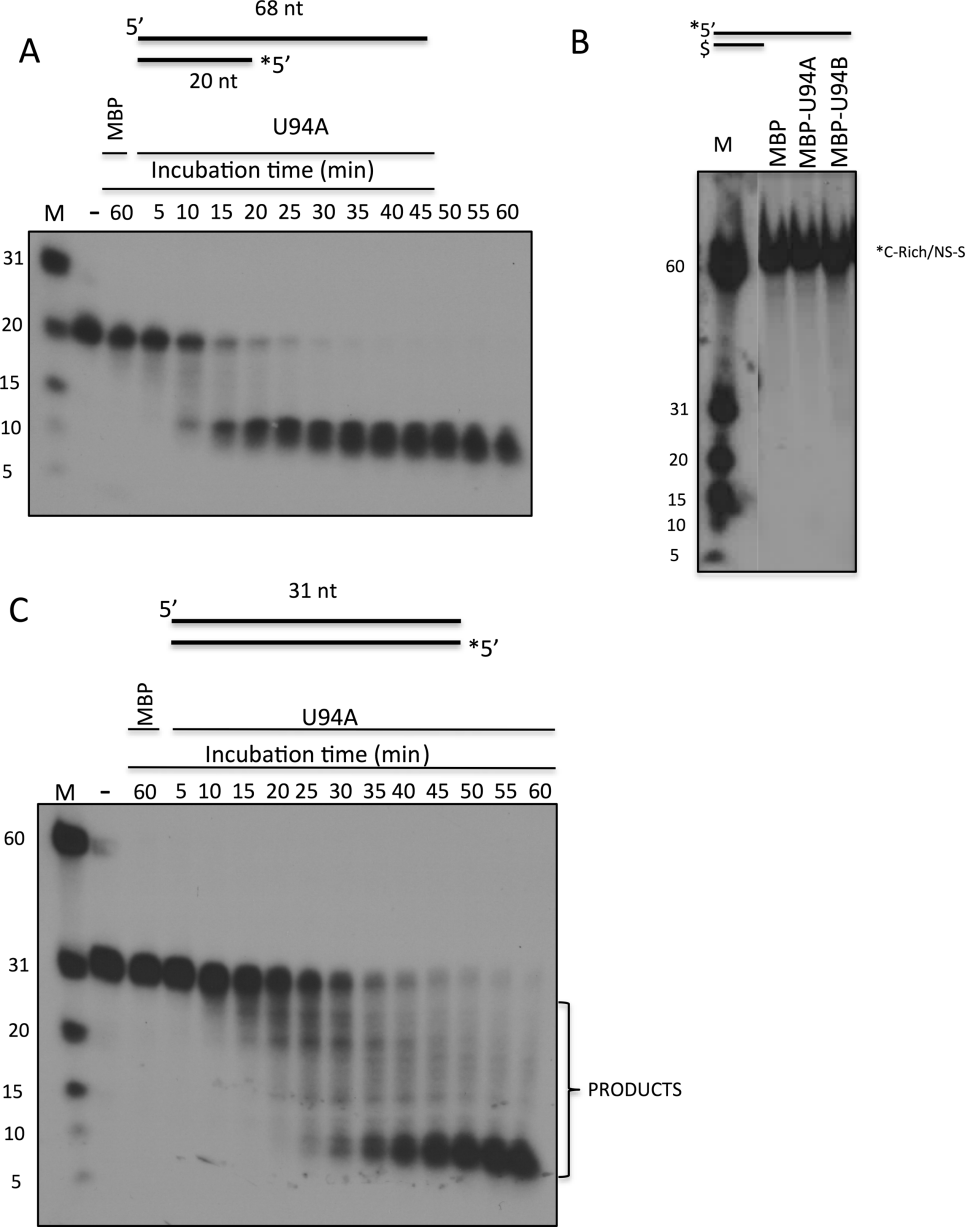
Characterization of HHV-6 U94 exonuclease activity. (**A**) Proteins were incubated for various periods of time (5–60 min) with a partial dsDNA duplex with the shorter strand being labeled at its 5′ end, identified by the asterisk (*). After incubation, loading buffer was added and samples boiled for 5 min at 95°C and immediately chilled on ice. Samples were separated by denaturing gel electrophoresis. Gels were dried and exposed to film. (**B**) Proteins were incubated with a partial dsDNA duplex in which the longer strand was 5′ end-labeled (*) and the short strand contained a phosphorotioate bond ($) at its 3′ end before the last nucleotide. After a 30-min incubation, samples were separated by denaturing gel electrophoresis. Gels were dried and exposed to film. (**C**) Same as in (A) except that the dsDNA duplex was blunt at both ends. Results are representative of two independent experiments.

We next determined the kinetics of the exonuclease activity and strands dissociation by MBP-U94A. Partial dsDNA (Figure 7A) and complete dsDNA templates were analyzed (Figure 7C). The DNA duplexes were incubated with MBP or MBP-U94A for various time periods ranging from 5 to 60 min. Strand displacement and exonuclease activity was rapidly detected with the MBP-U94 proteins, resulting in a shortening of the probe to 8–10 nt in length over time, while the MBP control protein did not display any activity after 60 min (Figure 7A). These results indicate that the dsDNA is attacked from its 3′ end and gradually digested by the U94 exonuclease activity. Once the DNA strand reaches 8–10 nt in length the two strands separate from each other, possibly through helicase activity of the enzyme. To determine if MBP-U94A is active on 3′ protruding ends, we generated a DNA substrate similar to that of Figure 7A except that the bottom strand was protected with a phosphorothioate bond at its 3′ end and that the top strand containing a 3′ protruding end was labeled at its 5′ extremity. This DNA substrate was not attacked by MBP-U94A or MBP-U94B (Figure 7B), demonstrating that these enzymes are not active on 3′ protruding ends. When a blunt dsDNA substrate was used, we observed various size intermediates including some full-length probes that remain intact over the late kinetic time points (Figure 7C). These results are compatible with dsDNA being attacked from both sides by U94.

### Importance of E27 for U94 exonuclease activity

**Figure 8.**
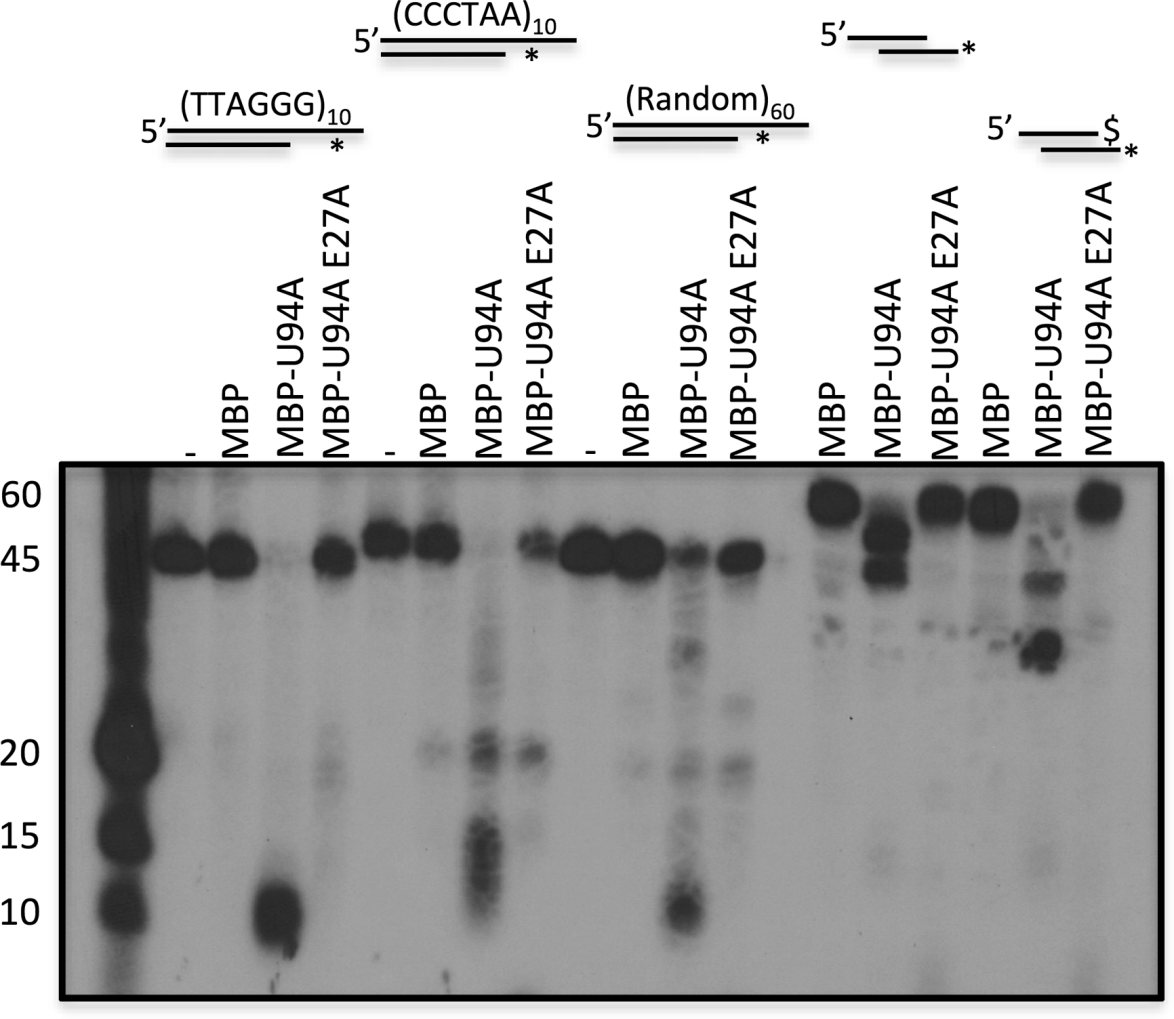
Characterization of HHV-6 U94 helicase/exonuclease activity. Proteins were incubated with various DNA substrates for 30 min at 37°C. The asterisk denotes the 5′ end-labeled strand and the $ symbol denotes phosphorotioate bonds. After incubation samples were either separated by denaturing. Gels were dried and exposed to film. Results are representative of two independent experiments.

U94 residues important for the 3′ to 5′ exonuclease activity could not be deduced from the alignment with Rep68 as this protein lacks such enzymatic activity. While searching for such enzymes we found that the Werner (WRN) exonuclease/helicase displayed some activities similar to U94. The N-terminal portion of WRN and amino acid E84 have been shown to be essential for the exonuclease activity of WRN (43). After analysis of U94 amino acid sequence, we identified the amino acid E27 as a potential active site for the exonuclease activity and mutated it to alanine to generate MBP-U94A E27A. The exonuclease activity of MBP-U94A and MBP-U94A E27A was tested on various DNA substrates. Since MBP-U94 preferentially binds telomeric motifs over unspecific DNA (Figure 3), we generated partial dsDNA substrates with telomeric motifs or random sequences. Both dsDNA substrates containing the telomeric motifs were efficiently processed by MBP-U94A with no intact probe remaining (Figure 8). Unlike the telomeric motifs that were completely processed, only a portion of the random dsDNA was digested/separated suggesting a higher processivity rate of U94 on telomeric dsDNA. The MBP-U94A E27A mutant was unable to process such substrates. We next studied the effects of MBP-U94A on a 39 bp dsDNA duplex containing two 3′ recessive ends. This DNA structure was simultaneously attacked by MBP-U94A from both 3′ ends yielding two major products of ∼50 and 40 nt. When the 3′ end of the upper strand was protected with a phosphorothioate bond, the non-protected strand was digested all the way down to the end of the upper strand, yielding a 25 nt product. Again, the MBP-U94A E27A mutant was very ineffective at processing such substrate.

### U94 prefers to attack 3′ recessed over blunt ends

**Figure 9.**
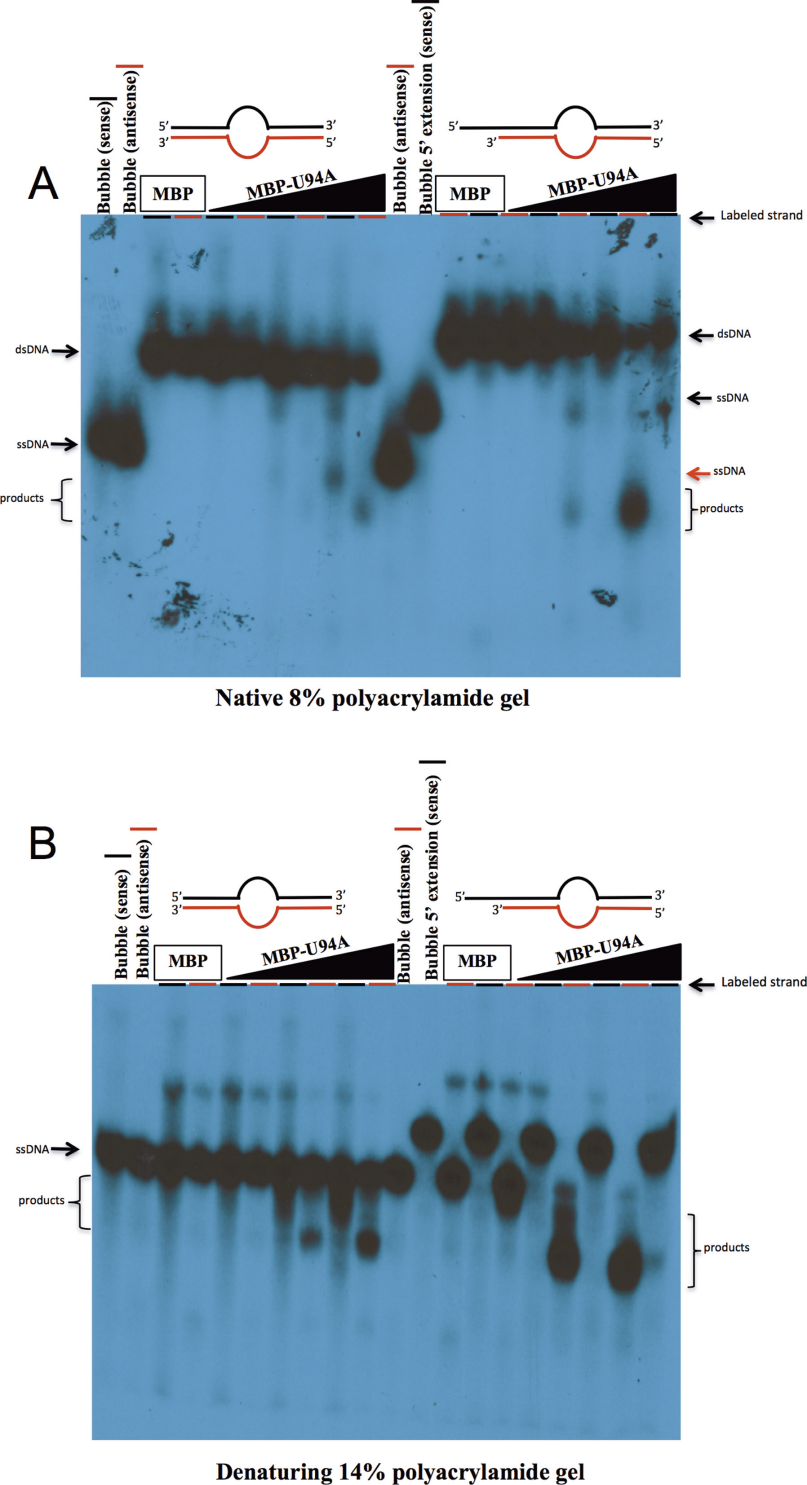
U94 prefers 3′ recessed ends over 3′ blunt ends. Strands of bubble DNA structure containing either blunt extremities (left portion of figure) or a 5′ protruding end and a blunt end (right portion of figure) were individually labeled and annealed with their non-labeled complimentary strands. The dsDNA duplexes were incubated with recombinant proteins for 30 min at 37°C in reaction buffer. Samples were separated by non-denaturing (**A**) or denaturing (**B**) gel electrophoresis. Gels were dried and exposed to film. For each lane, the labeled strand is indicated by the red or black line. Results are representative of two independent experiments.

From the results presented above, MBP-U94 is able to exert its 3′ to 5′ exonuclease activity against blunt and 3′ recessive ends but not over 3′ protruding ends. To determine which type of DNA ends and expand on the types of substrate U94 can process, we generated two bubble DNA structures that differed only in their termini to determine whether U94 prefers blunt or 3′ recessive ends. Either strand were 5′ end-labeled and the DNA was subjected to U94 exonuclease assay. The bubble DNA with two blunt ends was attacked equally on both sides by MBP-U94 (Figure 9A and B). By comparison, when a bubble with a blunt and a 3′ recessive end was used, exonuclease activity was only observed from the 3′ recessive end (Figure 9A and B).

### D-loop destabilization by U94

**Figure 10.**
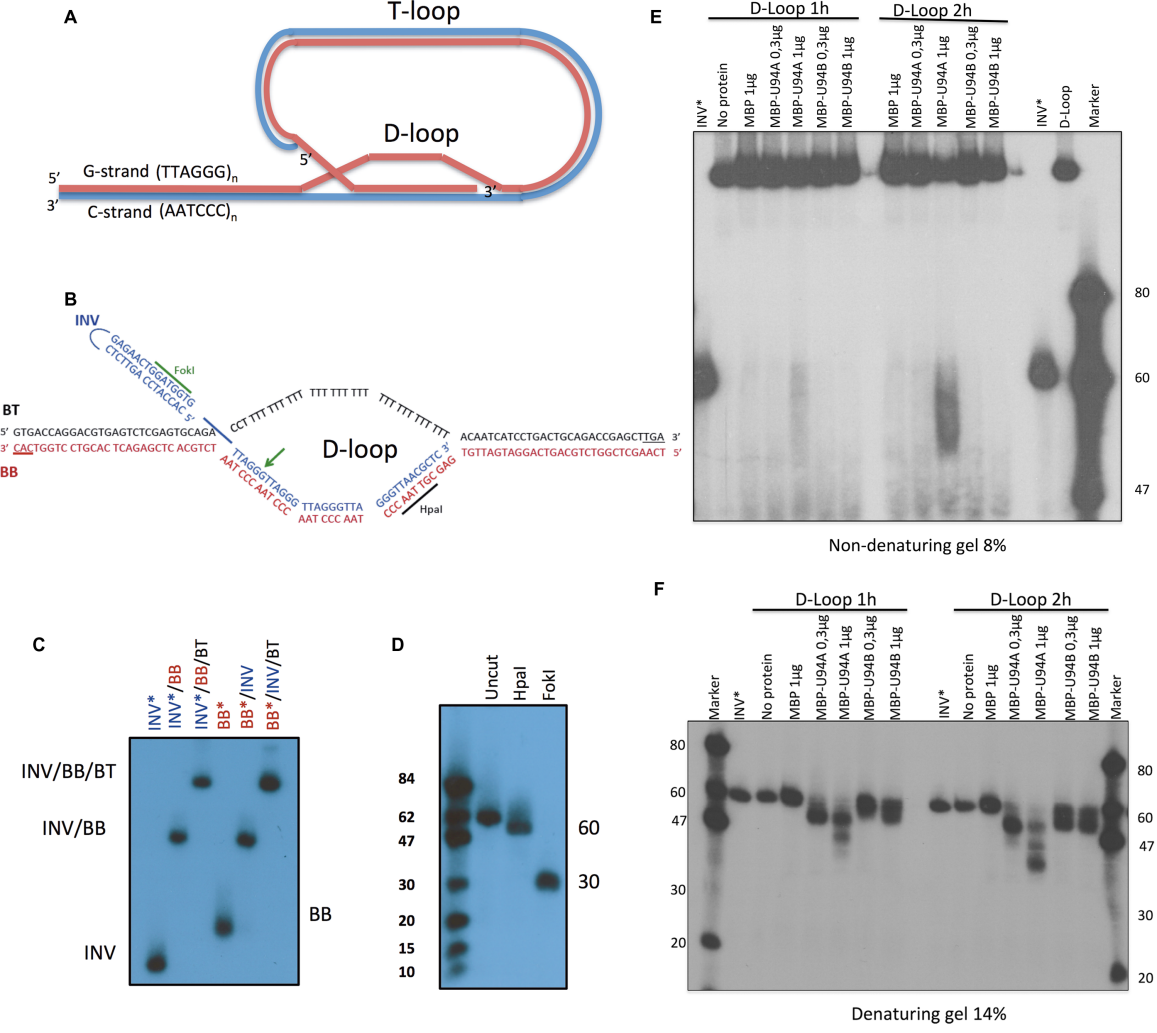
Telomeric D-loop destabilization by the HHV-6 U94 proteins. (**A**) Schematic structure of chromosome ends with T- and D-loop structures. (**B**) Structure and sequences of the oligonucleotides used to construct the synthetic telomeric D-loop. The 3′ ends of BB and BT oligos are protected with three phosphorotioate bonds. (**C**) Analysis of D-loop assembly by non-denaturing gel electrophoresis. The asterisk denotes the labeled strand. (**D**) Analysis of D-loop assembly (INV oligo is 5′ end-labeled) by HpaI and FokI restriction endonucleases. After digestion of the D-loop, the samples were boiled and analyzed by denaturing gel electrophoresis. (**E** and **F**) Assembled telomeric D-loop was incubated with proteins for 1 or 2 h after which the samples were separated by non-denaturing (E) or denaturing (F) gel electrophoresis. Gels were dried and exposed to film. Results are representative of two independent experiments.

Telomeres have a 3′ G-rich overhang that allows the formation of a T-loop by invasion of the double-stranded hexamer repeats. As the G-rich strand displaces one strand, a displacement loop (D) is created (48) (Figure 10A). The T-loop formation confers some protection from exonucleases and DNA damage responses. To determine if U94 is able to resolve this protective structure allowing recombination of the host telomeres with the viral TRS (reviewed in (8,9)), we determined whether U94 could attack the D-loop structure and compromise its integrity. We constructed a D-loop consisting of a bubble with two 33 bp duplex arms and a 33 bp-melted region in which an invading strand (INV) that mimics the 3′ telomeric tail (Figure 10B), as described (43). Analysis by native gel indicated that a D-loop with a 5′-end labeled INV or bottom strand (BB) yielded a single structure (Figure 10C). Restriction analysis confirmed the proper alignment (Figure 10D). We incubated MBP and MBP-U94 with the D-loop and analyzed the products on native (Figure 10E) and denaturing gels (Figure 10F). At the lowest doses used, the substrate was not sufficiently processed to release the INV strand. However, at the highest dose of MBP-U94A, we could detect bands migrating below the full-length INV strand (Figure 10E and F). Thus, the MBP-U94 exonuclease initiated digestion at the INV 3′ end and when sufficient digestion had occurred, the INV strand was released from the complex. These results suggest that the U94 helicase and exonuclease cooperate to resolve the telomeric D-loop, favoring recombination events.

## DISCUSSION

HHV-6A and HHV-6B have developed a mechanism that allows maintenance of the virus genome in infected cells by integrating their genetic material into the telomeres of host chromosomes. Based on estimates from several independent studies, ∼0.5–1% of world population harbor HHV-6 in every single cell of their body (iciHHV-6) (reviewed in (8–10,23)). This suggests that HHV-6 was able to infect a sperm cell or an ovum and integrate its genome into gametes at some point in time. Kaspersen *et al*. recently discovered that HHV-6A/B can be detected in 13% of sperm samples from healthy individuals, indicating that infected sperm cells could give rise to iciHHV-6 (49). Furthermore, HHV-6A/B appear to bind to the acrosome suggesting that it can be transported to the uterus and eventually to the ovum via sperm cells (49). Once integrated, every cell of the developing fetus will contain a copy of HHV-6A/B DNA. The 0.5–1% prevalence of iciHHV-6 therefore only represents the integration events that occur in gametes. Integration into somatic cells following primary infection is likely more frequent and would represent a mechanism allowing HHV-6A/B to establish latency. However, since these latently infected cells are few and hard to isolate from infected individuals, they are not detected using conventional techniques (9).

The integration mechanism of HHV-6A/B remains poorly understood. Another herpesvirus capable of integration is MDV, an oncogenic alphaherpesvirus that causes deadly lymphoma in chickens. The precise MDV integration mechanisms are unknown but it is surmised that the MDV gene encoding for an RNA telomerase subunit that shares 88% sequence identity with the chicken telomerase RNA gene plays a role in MDV genome integration by aiding in the generation of telomeric elongations at the ends of the viral genome as a prerequisite for integration (30,50). The TRS encoded by MDV have been shown to facilitate integration of the virus into host telomeres, a process that is crucial for efficient pathogenesis and tumor formation. Although HHV-6A/B also possess TRS at their genome termini, it remains unknown if these sequences are important for integration. A unique protein that has been proposed to facilitate HHV-6A/B integration is the *U94* gene product, U94. U94 shares a sequence homology with the AAV Rep68, a protein playing an essential role in AAV integration into human chromosomes (33). To mediate integration, Rep68 requires at least three basic activities: DNA-binding, endonuclease and helicase-ATPase activities. The amino acids or domains essential for these activities were previously defined (44–46) and have been identified in U94 based on the sequence homology (Figure 2). In the present work, we have characterized the biochemical properties of HHV-6A/B U94. Our results indicate the following: (i) U94 is a DNA-binding proteins. We could demonstrate that U94 is capable of binding ssDNA and dsDNA. Gel-shift experiments indicate that U94 binding to DNA can be efficiently competed with random DNA, suggesting that U94 binds nucleic acids irrespective of its DNA composition. To determine the affinity of U94 for different DNA molecules we performed SPR analyses. Using SPR, we could determine that HHV-6A/B U94 proteins have greater affinity for certain DNA molecules such as ssDNA molecules harboring CCCTAA telomere motifs. Furthermore, our results suggest that HHV-6A/B U94 can also efficiently bind dsDNA carrying human TRS motifs as well as the TRS sequences present at the HHV-6A/B genome termini. Binding to the ssDNA equivalent of the ssRNA TERC revealed some but minimal binding by U94 suggesting either that a minimum length is required or that more than one CCCTAA repeat is required for proper binding. Subsequent experiments indicated that three CCCTAA motifs were needed for maximal binding; (ii) HHV-6A/B U94 can hydrolyze ATP into ADP and to a lesser extent ADP into AMP. U94 ATPase activity is independent of the presence of DNA; (iii) HHV-6A/B U94 proteins display strong 3′ to 5′ exonuclease activity with 3′ recessive ends being the preferred substrates over blunt ends. No exonuclease activity on 3′ protruding or 5′ ends was observed. U94 exonuclease activity requires Mg^++^ and is inhibited by Ca^++^ or Zn^++^ ions; (iv) U94's have weak ATP-independent helicase activity. (v) No endonuclease activity could be monitored (not shown); (vi) U94's can efficiently attack telomeric D-loop structure and compromise its integrity.

U94 enzymatic activities are compatible with a possible role for these proteins during HHV-6A/B integration into host telomeres. We surmise that expression of U94 very early during infection (51,52) would compromise the integrity of the chromosomal ends by attacking the D-loop structure generating a partial single stranded DNA strand with the CCCTAA motif. Simultaneously, it can attack the 3′ ends of the linear viral genome generating 5′ protruding ends complimentary to the CCCTAA motifs. Annealing of the DNA molecules would result in the loss of the pac2 sequence of the DR_R_, as previously reported (53,54). The pac1 sequence from the other end of the viral genome is also missing and proposed to be lost through erosion during chromosomal replication (54). Telomeric viral repeats would then serve as template for telomerase and chromosome elongation (54). Homologous recombination was also proposed as a possible mechanism explaining HHV-6A/B integration (8,9). Further studies are needed, including the generation of a HHV-6 U94 deletion mutant to determine the relative contribution of this protein during the integration process.

The integration mechanisms of HHV-6A/B that allow insertion of the viral genome into telomeres of germ and somatic cells remain poorly understood and should be further addressed in the future. An equally important aspect of iciHHV-6 is whether these viruses can efficiently excise themselves from host DNA, resulting in the production of new infectious particles. Although several reports have described disease associations in individuals with iciHHV-6, these individuals appeared otherwise healthy in most cases. In a recent review cases of iciHHV-6 were tabulated and the prevalence of iciHHV-6 was 2.3× greater in diseased individuals relative to controls (10). This suggests that our immune system is able to control sporadic reactivation events and when our immunity diminishes, such as in transplant patients, HHV-6 reactivation occurs with possible sequelae (55). Endo *et al*. recently reported convincing *in vivo* evidence that under profound immunocompromised conditions (X-SCID), iciHHV-6A can excise itself from integration and cause disease in humans (56). These results corroborated previous findings that suggested reactivation and transplacental transmission of reactivated iciHHV-6 in pregnant women (57). Such results should prompt transplant clinicians to investigate the iciHHV-6 status of their patients as well as that of the organs to be transplanted.

In summary, we provided a detailed characterization of the biological and enzymatic activities of HHV-6A/B U94 proteins. These activities of U94 are compatible with a role for U94 in HHV-6A/B integration into the telomeres of host chromosomes and could also contribute to viral excision, which should be determined in future studies.

## SUPPLEMENTARY DATA

Supplementary Data are available at NAR Online.

SUPPLEMENTARY DATA
