# Supplementary material for: Characterization of human herpesvirus 6A/B U94 as ATPase, helicase, exonuclease and DNA-binding proteins
Source: Nucleic Acids Res. 2015 May 20;43(12):6084–98. doi: 10.1093/nar/gkv503 (PMC4499131; doi:10.1093/nar/gkv503)
Supplement: SUPPLEMENTARY DATA [file supp_43_12_6084__index.html]

Characterization of human herpesvirus 6A/B U94 as ATPase, helicase, exonuclease and DNA-binding proteins — Characterization of human herpesvirus 6A/B U94 as ATPase, helicase, exonuclease and DNA-binding proteins — SUPPLEMENTARY DATA 

# Characterization of human herpesvirus 6A/B U94 as ATPase, helicase, exonuclease and DNA-binding proteins

## SUPPLEMENTARY DATA

- SUPPLEMENTARY DATA
