# Supplementary material for: Characterization of human herpesvirus 6A/B U94 as ATPase, helicase, exonuclease and DNA-binding proteins
Source: Nucleic Acids Res. 2015 May 20;43(12):6084–98. doi: 10.1093/nar/gkv503 (PMC4499131; doi:10.1093/nar/gkv503)
Supplement: SUPPLEMENTARY DATA [file supp_gkv503_Supplementary_table_and_figures.pdf]

Supplementary Table 1 : List of oligonucleotides used in this study.		Figure
**Gel shift assay**		
Telomeric G-Rich	5'-GG(TTAGGG)$_7$TTAG- 3'	2
Random sequence	5'- GGTATCGGTATCGGTATCGGTATCGGTATCGGTATCGGTATCGGTATC -3'	2-3
Human TRS	5'- GG(TTAGGG)$_7$TTAG -3'<br>3'- CC (AATCCC)$_7$AATC -5'	3
**Surface plasmon resonance assay**		
ΔITR AAVS1 Rep binding site	5'- GATCAGTGATGGAGTTGGCCACTCCCTCTCTGCGCGCTCGCTCGCTCACTGAGGCC/Bio/ -3'<br>3'-TCACTACCTCAACCGGTGAGGGAGAGACGCGCGAGCGAGCGAGTGACTCCGGGATC -5'	4A
Telomeric G-rich	5' /Bio/GG(TTAGGG)$_7$TTAG- 3'	4A-4B
Telomeric C-rich	5' /Bio/C(TAACCC)$_7$TAACC - 3'	4A-4B
HHV-6 TRS C-rich	5'- C(TAACCC)$_3$TAGGCCC(TAACCC)$_2$TAGGTCTAACCCT /Bio/ -3'	4A-4B
Random sequence	5'- /Bio/GGTATCGGTATCGGTATCGGTATCGGTATCGGTATCGGTATCGGTATC -3'	4A-4B
HHV-6 TRS	5'- CTAACCCTAACCCTAACCCTAGGCCCTAACCCT AACCCTA GGTCTAACCCT/ Bio/ -3'<br>3'- ATTGGGATTGGGATTGGGATCCGGGATTGGGATTGGGATCCAGATTGGGAC -5'	4A-4B
TERC DNA equivalent	5'-Bio/GTGGCCATTTTTTGTCTAACCCTAACTGAGAAGGGCGTAG -3'	4C
Human TRS C-rich	5' /Bio/C(TAACCC)$_7$TAACC - 3'	4C
Human TRS	5'- /Bio/GG(TTAGGG)$_7$TTAG -3'<br>3'- CC(AATCCC)$_7$AATC-5'	4C
**Exonuclease/Helicase assay**		
NS probe	5'- AATGTATCGACATCCAGCGA -3'	
C-rich/NS probe at 3' end	5'- CCCTAACCCTAACCCTAACCCTAACCCTAACCCTAACCCTAACCCTAATCGCTGGATGTCGATACATT-3'<br>3'-AGCGACCTACAGCTATGTAA-5'	6A
C-rich NS probe in middle	5'- TAACCCTAACCCTAACCCTAACCCTCGCTGGATGTCGATACATTTAACCCTAACCCTAACCCTAACCC-3'<br>3'- AGCGACCTACAGCTATGTAA -5'	6B
C-rich/NS probe at 5' end	5'- TCGCTGGATGTCGATACATTCCCTAACCCTAACCCTAACCCTAACCCTAACCCTAACCCTAACCCTAA -3'<br>3'- AGCGACCTACAGCTATGTAA -5'	6C-7A-7B-8A-8B
Blunt DNA	5'- ATGCATGCATTTAGGGTTAGGGTTAGGGTTA-3'<br>3'- TAACCCTAACCCTAACCCTAAATGCATGCAT-5'	8C
(TTAGGG)$_{10}$	5'-AGCTGAGCATGTCCAGGGTTAGGGTTAGGGTTAGGGTTAGGGTTAGGGTTAGGGTTAGGG-3'<br>3'-TCGACTCGTACAGGTCCCAATCCCAATCCCAATCCCAATCCCAAT-5'	9
(CCCTAA)$_{10}$	5'-AGCTGAGCATGTCCACCCTAACCCTAACCCTAACCCTAACCCTAACCCTAACCCTAACCC-3'<br>3'-TCGACTCGTACAGGTGGGATTGGGATTGGGATTGGGATTGGGATT-5'	9
(Random)$_{60}$	5'- AGCTGAGCATGTCCAAGCTGAGCATGTCCAAGCTGAGCATGTCCAACCACACTCAGATCT-3'<br>3'- TCGACTCGTACAGGTTCGACTCGTACAGGTTCGACTCGTACAGGT-5'	9
Partial dsDNA	5'-CCCTAACCCTAACCCTAACCAAATTTAAAGGGTTTAAAGGGTTTAAATTTAAATTTAAA-3'<br>3'-<br>TTTAAATTTCCCAAATTTCCCAAATTTAAATTTAAATTTCCAATCCCAATCCCAATCCC-5'	9
Bubble blunt	5'-GCGCGGAAGCTTGGCTGCAgaatattgCTAGCGGGAATTCGGCGCG-3'<br>3'-CGCGCCTTCGAACCGACGTtccgcggtGATCGCCCTTAAGCCGCGC-5'	10
Bubble 3' recessed	5'-TATGATCTCATGTCGCGCGGAAGCTTGGCTGCAgaatattgCTAGCGGGAATTCGGCGCG-3'<br>3'-GCCTTCGAACCGACGTtccgcggtGATCGCCCTTAAGCCGCGC-5'	10
INV	5'-CACCATCCAGTTCTCTTTTGAGAACTGGATGGTGTTAGGGTTAGGGTTAG GGTTAGGGTTAACGCTC-3'	11
BB	5'AAGCTCGGTCTGCAGTCAGGATGATTGTGAGCGTTAACCCTAACCCTAACCCTAACCCTAATCTGCA-CTCGAGACTCACGTCCTGGTCA$C-3'	11
BT	5'GTGACCAGGACGTGAGTCTCGAGTGCAGACCTTTTTTTTTTTTTTTTTTTTTTTTTTTTTTACAATCAT-CCTGACTGCAGACCGAGCT$T-3'	11

Underline denotes double-stranded regions. Sequences in lower case represent non-complimentary regions. $-denotes a phosphorothioate bond.

Figure S1: U94 and Rep68 protein sequence alignment.

```
U94A           MFSIINPSDDFWTKDKYIMLTIKGPMEWEAEIPGISTDFFCKFSN--VSVPHFRDMHSPG 58
U94B           MFSIINPSDDFWTKDKYIMLTIKGPVEWEAEIPGISTDFFCKFSN--VPVPHFRDMHSPG 58
Rep68          -------------MPGFYEIVIKVPSDLDEHLPGISDSFVNWVAEKEWELPPDSDMDLN- 46
                               :  :.** * : : .:**** .*.  .::    :*   **.

U94A           APDIKWITACTKMIDVILNYWNNKTAVPTPAKWYAQAENKAGRPSLILLIALDGIPSATI 118
U94B           APDIKWITACTKMIDVILNYWNNKTAVPTPAKWYAQAENKAGRPSLTLLIALDGIPTATI 118
Rep68          LIEQAPLTVAEKLQRDFLTEWRRVSKAP-EALFFVQFEKGESYFHMHVLVETTGVKSMVL 105
                 :   :*.. *:   :*. *.. : .*  * ::.* *:  .   : :*:   *: : .:

U94A           GKHTTEIRGVLIKDFFDGNAPKIDDWCTYAKTKK-NGGGTQVFSLSYIPFALLQIIRPQF 177
U94B           GKHTTEIRGVLIKDFFDGNAPKIDDWCTYAKTKK-NGGGTQVFSLSYIPFALLQIIRPQF 177
Rep68          GRFLSQIREKLIQRIYRGIEPTLPNWFAVTKTRNGAGGGNKVVDECYIPNYLLPKTQPEL 165
               *:. ::**  **: :: *  *.: :* : :**::  ***.:*.. .***  **   :*::

U94A           QWAWTNINELGDVCDEIHRKHIISHFNKKPNVKLMLFPKDGIN--------GISLKSKFL 229
U94B           QWAWTNINELGDVCDEIHRKHIISHFNKKPNVKLMLFPKDGTN--------RISLKSKFL 229
Rep68          QWAWTNMEQYLSACLNLTERKRLVAQHLTHVSQTQEQNKENQNPNSDAPVIRSKTSARYM 225
               ******:::  ..* :: .:: :   :.   :     *:. *          . .::::

U94A           GTIEWLSDLGIVTEDAWIRRDIRSYMQLLTLTHGDVLIHRALSIAKKRIRATRKAIDFIA 289
U94B           GTIEWLSDLGIVTEDAWIRRDVRSYMQLLTLTHGDVLIHRALSISKKRIRATRKAIDFIA 289
Rep68          ELVGWLVDKGITSEKQWIQEDQASYISFNAASNSRSQIKAALDNAGKIMSLTKTAPDYLV 285
                 : ** * **.:*. **:.*  **:.: ::.   *: **. : * :  *:.* *::.

U94A           HIDTDFQIYENPVYQLFCLQSFDPILAGTILYQWLSHRGGKKNTVSFIGPPGCGKSMLTG 349
U94B           HIDTDFEIYENPVYQLFCLQSFDPILAGTILYQWLSHRRGKKNTVSFIGPPGCGKSMLTG 349
Rep68          GQQPVEDISSNRIYKILELNGYDPQYAASVFLGWATKKFGKRNTIWLFGPATTGKTNIAE 345
                 :.  :* .* :*::: *:.:**  *.:::  * ::: **:**: ::**.  **: ::

U94A           AILENIPLHGILHGSLNTKNLRAYGQVLVLWWKDISINFDNFNIIKSLLGGQKIIFPINE 409
U94B           AILENIPLHGILHGSLNTKNLRAYGQVLVLWWKDISINFENFNIIKSLLGGQKIIFPINE 409
Rep68          AIAHTVPFYGCVNWTNENFPFNDCVDKMVIWWEEGKMTAKVVESAKAILGGSKVRVDQKC 405
               ** ..:*::* :: : :.  :.   : :*:**:: .:. . .:  *::***.*: .  :

U94A           NDHVQIGPCPIIATSCVDIRSMVHSN----LHKINLSQRVYNFTFDKVIPRNFPVIQKDD 465
U94B           NDHVQIGPCPIIATSCVDIRSMVHSN----IHKINLSQRVYNFTFDKVIPRNFPVIQKDD 465
Rep68          KSSAQIDPTPVIVTSNTNMCAVIDGNSTTFEHQQPLQDRMFKFELTRRLDHDFGKVTKQE 465
               :. **.* *:*.** .:: :::..*     *:  *.:*:::* : : : ::*  : *::

U94A           INQFLFWARNRSINCFIDYTVPKIL----------------------------------- 490
U94B           INQFLFWARNRSINCFIDYTVPKIL----------------------------------- 490
Rep68          VKDFFRWAKDHVVEVEHEFYVKKGGAKKRPAPSDADISEPKRVRESVAQPSTSDAEASIN 525
               :::*: **::: ::   ::  * *

U94A           ------------------------------------------------------------
U94B           ------------------------------------------------------------
Rep68          YADRYQNKCSRHVGMNLMLFPCRQCERMNQNSNICFTHGQKDCLECFPVSESQPVSVVKK 585


U94A           ------------------------------------
U94B           ------------------------------------
Rep68          AYQKLCYIHHIMGKVPDACTACDLVNVDLDDCIFEQ 621
```

Figure S1: Amino acids required for biochemical activities of Rep68 (DNA binding, helicase, ATPase, endonuclease) are colored in **red**. **Black** frames delimit required domains of the helicase activity. Active residue of helicase activity is indicated in **blue**. Identical amino acid between sequences are represented by an asterisk **(*)** Conserved amino acids by a colon **(:)** Semi-conserved by a period **(.)**

Figure S2 : MBP fusion proteins production.

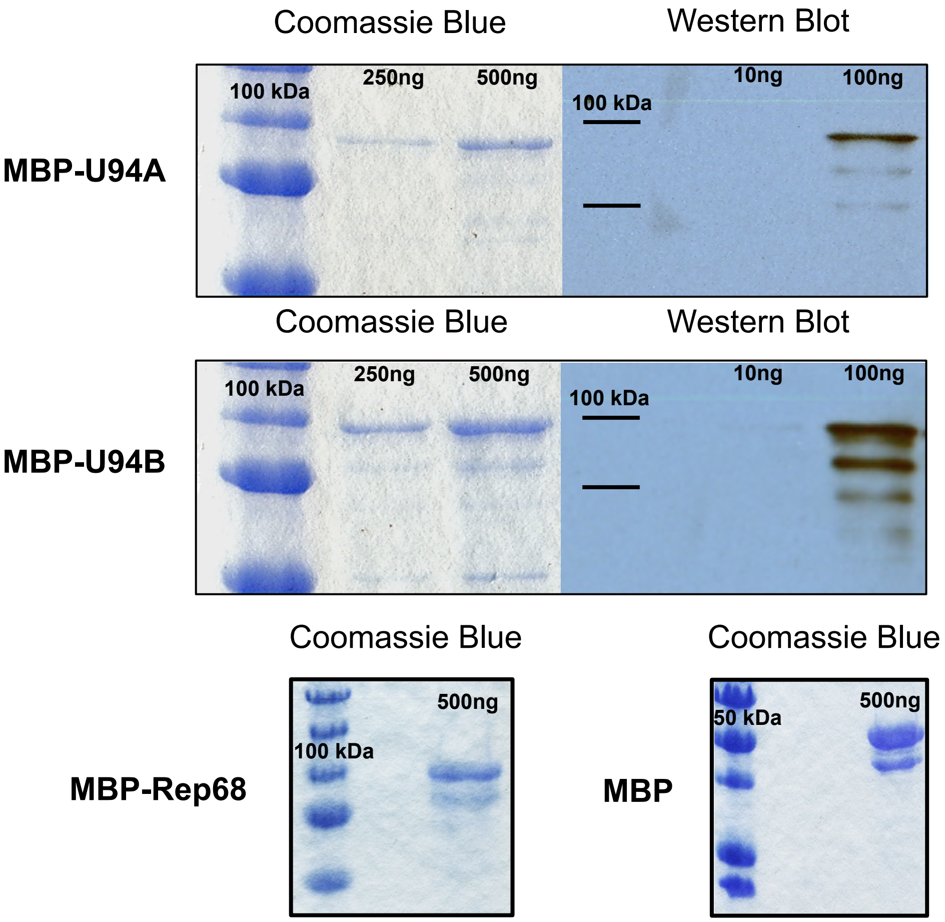

Figure S2 : Analysis of purified MBP and MBP fusion proteins by Coomassie blue staining and western blot. For detection by western blot, rabbit anti-MBP (1:1000) and goat anti-rabbit antibodies coupled with peroxidase (1:10000) were used.
